# Handheld transabdominal ultrasound, after limited training, may confirm first trimester viable intrauterine pregnancy: a prospective cohort study

**DOI:** 10.1080/02813432.2021.1910643

**Published:** 2021-04-14

**Authors:** Judith Krossøy Pedersen, Cecilie Sira, Jone Trovik

**Affiliations:** a Department of Clinical Science, University of Bergen, Bergen, Norway; b Department of Obstetrics and Gynaecology, Haukeland University Hospital, Bergen, Norway

**Keywords:** Diagnostics, family medicine, handheld ultrasound, point-of-care ultrasound systems (POCUS), pregnancy, threatening miscarriage

## Abstract

**Objectives:**

Handheld point-of-care abdominal ultrasound (POCUS) may be used by primary care physicians while vaginal ultrasound is limited to use in specialist care. We aimed to compare abdominal handheld ultrasound to vaginal ultrasound in determining first trimester viable intrauterine pregnancy and estimate gestational length.

**Design:**

Prospective cohort study.

**Setting:**

Gynaecologic outpatient clinic; women referred from GPs during early pregnancy. Handheld ultrasound using VscanExtend^®^ was performed by fourth-year medical students with limited training. Transvaginal ultrasound using high-end devices was performed by ordinary hospital staff.

**Subjects:**

Women in the first trimester of pregnancy referred for termination of pregnancy or with symptoms of early pregnancy complications.

**Main outcome measures:**

Rate of confirming vital intrauterine pregnancy (visualizing foetal heart beats) and measurement of crown-rump length (CRL) using handheld abdominal versus vaginal ultrasound.

**Results:**

In all 100 women were included; 86 confirmed as viable intrauterine pregnancies and 14 pathological pregnancies (miscarriages/extrauterine pregnancies). Handheld abdominal ultrasound detected fetal heartbeats in 63/86 (73% sensitivity) of healthy pregnancies and confirmed lack of fetal heartbeats in all pathological pregnancies, total positive predictive value (PPV) 100% and total negative predictive value (NPV) 38%. From gestational week 7, handheld abdominal ultrasound confirmed vitality in 51/54 patients: PPV 100% and NPV 79%. CRL (*n* = 62) was median 1 mm shorter (95% confidence interval 1–2 mm) measured by handheld abdominal versus vaginal ultrasound.

**Conclusion:**

Handheld ultrasound has an excellent prediction confirming viable intrauterine pregnancy from gestational week 7. Validation studies are needed to confirm whether the method is suitable in primary care assessing early pregnancy complications.

**KEY POINTS**
- When early pregnancy vitality needs to be confirmed, women will traditionally be referred to secondary care for transvaginal comprehensive ultrasonography performed with high-end devices by imaging specialists.
- In this study personnel with limited former training (fourth-year medical students) performed transabdominal POCUS using a handheld device, investigating 100 first trimester pregnancies for confirmation of viability.
- Using handheld ultrasound viable pregnancy was confirmed from gestational week 7 with 79% positive and 100% negative predictive value.
- If handheld ultrasound used in primary care confirms vital intrauterine pregnancy, the need for specialist referral could be reduced.

## Introduction

Acute bleeding or pain in early pregnancy is relatively common and occurs in 20–25% of recognized pregnancies before week 20 [1,2]. This will often cause concern for the woman and her physician. Nearly half of all women experiencing bleeding in early pregnancy will have a miscarriage [1–3]. Vaginal ultrasound is considered the gold standard in assessing early pregnancy, confirming viable intrauterine pregnancy by visualizing intrauterine foetal heartbeats (FHB+). First trimester ultrasound measuring the foetal crown-rump length (CRL) also estimates gestational length more precisely than using the first day of the last menstrual period (Naegele’s rule) [4–6]. A vaginal ultrasound will rarely be available in primary health care, thus necessitating referral to a gynaecologist or hospital care.

Point-of-care ultrasound (POCUS) is bedside ultrasound performed by competent doctors, but not necessarily specialists for the given field, for example, gynaecology or radiology, using either high-end apparatuses or smaller handheld devices.

Handheld ultrasound is a small device that is easily transported in the pocket [7] and is more available in primary care, due to a lower cost. Studies have shown good overall agreement between handheld versus more high-end devices. Given a certain level of pre-test probability, POCUS can be safely used in a wide range of clinical settings [7]. In primary care, the most common indications for use of POCUS are abdominal, obstetric, and cardiac examinations [8]. A recent Scandinavian Delphi process identified first-trimester ultrasound examination among the top three investigations considered essential for GPs during daily work and recommended for a basic ultrasound curriculum [9]. Studies have shown good agreement between ultrasound examinations performed in primary care centres compared to ultrasound examinations in specialist care [7,8,10], including the obstetric and gynaecological field [11]. The best results by using POCUS in primary care is seen in settings where examinations, after an individually customized amount of training, are focused rule-in tests in patients with a high pre-test probability rather than screening-based investigations [7,8]. This gives few false positives and limits the rate of random findings [8]. The use of POCUS may lead to more efficient diagnostics [7,10] and is likely more cost-effective than ultrasound in secondary health care. The use of POCUS has increased over the last few years, and in 2016 30% of Norwegian GPs used ultrasound, however, three out of four GPs performed less than 10 scans annually [12].

If early pregnancy viability could be confirmed by the GP in situations with pregnancy complications (bleeding, pain, abrupt reduction of pregnancy symptoms), the woman’s anxiety may be reduced [1,3] and health care kept at the primary care level. There are, however, limited studies evaluating point-of-care handheld ultrasound during the first trimester of pregnancy [11,13]. To the best of our knowledge, none have specifically addressed the applicability specified per gestational weeks comparing use of handheld abdominal ultrasound to a vaginal ultrasound.

Our primary aim was to investigate if handheld abdominal ultrasound could detect viable intrauterine pregnancy during the first trimester and establish the lower limit of gestational length for a reliable vitality confirmation. Our secondary aim was to compare the CRL measured by handheld abdominal versus vaginal ultrasound.

## Materials and methods

We have performed a prospective cohort study, comparing the use of abdominal handheld POCUS ultrasound to high-end transvaginal ultrasound assessing first trimester pregnancies.

The study population consisted of pregnant women referred from primary health care for investigation at the gynaecologic outpatient department Haukeland University Hospital, Bergen, Norway from 28 March 2018 through 05 December 2018. The indication for early/first trimester investigation was either to determine vitality and gestational length as part of the clinical routine before pregnancy termination, to investigate suspected pregnancy complications: bleeding, pelvic pain, abrupt decreasing pregnancy symptoms, hyperemesis gravidarum, or after a previous pathological pregnancy to confirm an ongoing viable intrauterine pregnancy.

The aim was the inclusion of a minimum of 10 women pregnant in each of the gestational weeks 6 through 8, 30 examinations of pregnancies week 9 to 12, and 10 women with documented nonviable pregnancy.

The two different cohorts were established as follows. The ‘viable pregnancy group’ was defined as women with viable intrauterine pregnancies confirmed as visualized foetal heart beats (FHB+) by vaginal ultrasound. The ‘pathological pregnancy group’ was defined as women where no viable intrauterine pregnancy was detected, either an ongoing/completed miscarriage or extrauterine pregnancy. The rate of correctly identifying a pregnancy as vital (FHB+, belonging to the ‘viable pregnancy group’) or as not-vital (FHB−, belonging to the ‘pathological pregnancy group’) was compared using transabdominal POCUS performed on handheld devices versus transvaginal vaginal ultrasound using high-end apparatus.

Exclusion criteria were women not willing or able to give written consent.

After receiving consent, clinical information was collected regarding the woman’s age, date of last menstrual period (LMP), height and weight (computing body mass index, BMI). Women were first examined using handheld ultrasound (Vscan Extend^®^, GE Healthcare, Trondheim, Norway). Applying ultrasound gel above the symphysis, and then angling the sector-probe to visualize the uterus, the gestational sac and the foetus were identified. CRL was determined as the longest of three consecutive measurements of the foetal pole (the longest straight line of the foetus, from the outer margin of the cephalic pole to the rump). Vitality was noted as positive (+) when FHB was seen and as negative (−) if not definitely visualized. Three examiners have performed the handheld abdominal POCUS examinations; JT with >20 years of experience of gynaecologic ultrasound while JKP and CS were fourth-year medical students trained by JT during the study pilot phase and thereafter performed most of the examinations, and mostly in collaboration.

After the study ultrasound the patient’s scheduled gynaecological examination, including transvaginal ultrasound, was performed in another room by ordinary hospital staff (nurses trained in first trimester ultrasound or fellows training for gynaecology and obstetrics) using high-end stationary ultrasound apparatus, blinded for the prior study findings. This investigation determined the final diagnosis and allocated it to the viable pregnancy group or the pathological pregnancy group accordingly. CRL from the routine measurement was used to determine gestational length for the viable pregnancies using the Fetocalc^®^, Bergen, Norway according to Robinson [5]. When CRL was not measurable the gestational length was calculated according to LMP using Naegele’s rule [14].

To determine interrater variation in confirming viable pregnancy a subset of participants (*n* = 19) were subjected to a video recording of the ultrasound examination performed by one of the examiners and then the other examiners, based on the video, blinded and independently identified FHB + or FHB−.

All data were anonymized prior to analysis and stored on an approved electronic platform.

Using Statistical Packages for Social Sciences (SPSS) version 25, IBM. Chicago, USA, categorical variables (FHB+/FHB−) were compared by Chi-squared test or Fisher’s exact test (when cells with expected numbers <5). Continuous variables were compared by parametric or non-parametric tests as appropriate. For pairwise comparisons of continuous variables pairwise tests were used. Logistic regression was used to investigate whether gestational length or BMI influenced the detection rate by handheld abdominal POCUS. The interrater correlation was performed according to Fleiss Kappa statistics. All tests were two-sided and a *p*-value <0.05 was considered statistically significant.

Prior to starting the study ethical approval was granted from the Regional Ethics Committee (REK 2017/2030). All women gave written consent before inclusion.

## Results

In all 100 women were included; 86 referred with presumed vital pregnancies, mostly women seeking termination of pregnancy (*n* = 51) or hospitalized due to hyperemesis gravidarum (*n* = 21). Of these, six women (7%) were diagnosed with a pathological (non-vital) pregnancy after examination in the clinic (using routine high-end transvaginal ultrasound). For 14 women pregnancy pathology was suspected at referral due to vaginal bleeding, pain or abrupt decreasing pregnancy symptoms. Of these, six women (43%) were confirmed with a viable intrauterine pregnancy after examination by transvaginal ultrasound. The final ‘viable pregnancy group’ thus consisted of 86 women and the ‘pathological pregnancy group’ of 14 women with confirmed miscarriage (*n* = 10) or ectopic pregnancy (*n* = 4). Table 1 displays characteristics of the whole cohort and compares the viable pregnancy group and the pathological pregnancy group. The missing data regarding CRL measurements were mostly from the pregnancies of very low gestational length (5 weeks) with a too-small foetus to measure CRL even with transvaginal ultrasound.

**Table 1.** Comparisons of characteristics for the viable pregnancy group (confirmed viable intrauterine pregnancy) and the pathological pregnancy group (nonviable pregnancy).

	Viable pregnancy group		Pathological pregnancy group^b^		*p*-Value
	*n*^a^ = 86		*n* = 14		Mann–Whitney
	Median	95% CI^c^	Median	95% CI	
Age (years)	29	27–30	31	26–38	0.267
BMI^d^ (kg/m^2^)	23.4	22.1–24.5	25.6	19.5–29.4	0.432
Gestational length (days)	57^e^	54–60	55	48–93	0.901
	56^f^	52–62			
CRL^g^ vaginal (mm)	15	13–21			
CRL POCUS^h^ (mm)	16	12–20			

^a^*n* = numbers; ^b^*n* = 4 extrauterine pregnancy, *n* = 10 miscarriage; ^c^CI = confidence interval; ^d^BMI = body mass index, *n* = 31 missing data; ^e^calculated by Naegele’s rule; ^f^calculated using crown-rump length measured by transvaginal ultrasound; ^g^CRL = crown-rump length measured by transvaginal ultrasound, *n* = 11 no measurable CRL; ^h^CRL measured using handheld abdominal point-of-care ultrasound (POCUS), *n* = 23 missing data.

In Table 2 the vital intrauterine detection rate by handheld abdominal POCUS versus transvaginal ultrasound is specified according to gestational length. Although gestational week 5 initially was considered too early in pregnancy to identify FHB+, handheld abdominal POCUS confirmed FHB + in two of these 16 (13%) and transvaginal ultrasound 6/16 (38%), not significantly different, indicating that both methods have difficulty in identifying FHB + during week 5. Detection rates were not significantly different for any week group except for week 6 where handheld abdominal POCUS detected 10/16 (63%) compared to 16 (100%) detected by vaginal ultrasound, *p* = 0.018 (Fisher’s exact test) (Table 3). From week 7 and above (*n* = 54) handheld abdominal POCUS detected 51/54 91%, *p* = 0.243. None in the pathological pregnancy group were (wrongly) identified as FHB + neither by transvaginal ultrasound nor handheld abdominal POCUS.

**Table 2.** Comparison of visualization of heartbeats (FHB+) using handheld abdominal versus transvaginal ultrasound for different gestational weeks.

Gestational length	FHB+^a^; POCUS^b^	FHB+; Vaginal US^d^	*p*-Value, Fisher’s exact test
	*n*^c^ (%)	*n* (%)	
5 weeks, *n* = 16	2 (13)	6 (38)	0.125
6 weeks, *n* = 16	10 (63)	16 (100)	0.018
7 weeks, *n* = 13	13 (100)	13 (100)	1
8 weeks, *n* = 14	13 (93)	14 (100)	1
≥9 weeks, *n* = 27	25 (93)	27 (100)	0.491
Viable pregnancy group^e^, *n* = 86	63 (73)	76 (88)	0.019
The pathological pregnancy group^f^, *n* = 14	0 (0)	0 (0)	

^a^FHB+ = visualized fetal heartbeat; ^b^POCUS = handheld abdominal point-of-care ultrasound; ^c^*n* = numbers; ^d^vaginal US = vaginal ultrasound; ^e^confirmed viable intrauterine pregnancy; ^f^nonviable pregnancy (miscarriage or extrauterine pregnancy).

**Table 3.** Calculated sensitivity, specificity, positive and negative predictive value in identifying foetal heartbeats for 100 first trimester pregnancies using handheld abdominal ultrasound, specified for the earliest gestational weeks and for the whole study population.

	Handheld abdominal POCUS^a^			
	Sensitivity (%)	Specificity (%)	Positive predictive value (%)	Negative predictive value (%)
5 weeks^b^	13		100	
6 weeks	63	100	100	33
≥6 weeks	87	100	100	61
≥7 weeks	94	100	100	79
Total	73	100	100	38

^a^POCUS = point-of-care handheld ultrasound; ^b^missing calculations is due to lack of any of the 14 pathological pregnancies (miscarriage/extrauterine pregnancy) with gestational length week 5.

During week 6 handheld abdominal POCUS yielded a sensitivity of 63% in detecting vitality while the negative predictive value was 33%, demonstrating that a positive finding confirms a vital pregnancy, but a negative finding cannot confirm that the pregnancy is pathological. From week 7, the sensitivity was excellent: 94% in confirming vitality and a negative predictive value of 79% in confirming pathological pregnancy. Regarding transvaginal ultrasound, the sensitivity and specificity were 100% for each week from 6 and above, while a 38% sensitivity in week 5 led to an overall sensitivity of 88% percent.

Using logistic regression, higher gestational length (according to Naegele’s rule) was identified with an Odds Ratio of 1.05 (95% CI 1.02–1.09, *p* = 0.004) for detection of viable intrauterine pregnancy by handheld abdominal POCUS. BMI investigated as a linear factor had no impact on detection rate, Odds Ratio 1.03 (95% CI 0.91–1.17, *p* = 0.640).

**Figure 1.**
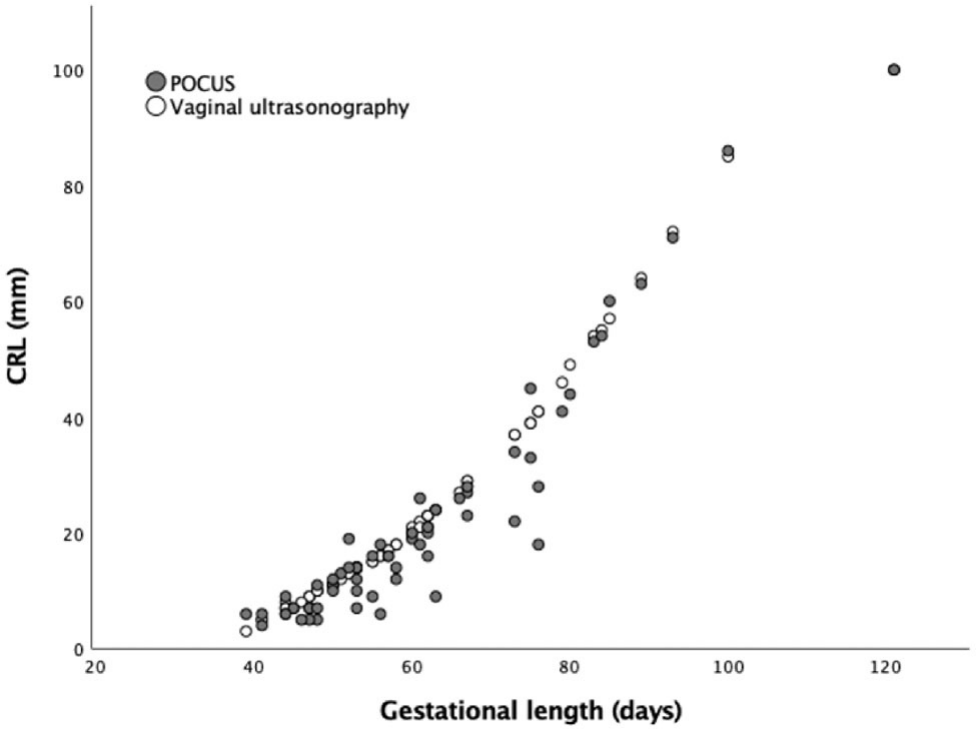
Scatterplot visualization of CRL measurements using handheld abdominal POCUS and transvaginal ultrasound for 62 women with viable intrauterine pregnancies. CRL: crown-rump length; POCUS: point-of-care ultrasound.

CRL was measured with both handheld abdominal POCUS and transvaginal ultrasound for 62 women (Table 4, Figure 1). For 7 of 62 women, the measurement was identical by both methods. Overall, the CRL was measured median 1 mm shorter by handheld abdominal POCUS than by transvaginal ultrasound.

**Table 4.** Differences in CRL^a^ measurements between handheld abdominal POCUS^b^ and transvaginal ultrasound for 62 viable first trimester pregnancies.

	Median	95% CI^c^	Minimum	Maximum	*p*-Value, Wilcoxon Signed Rank test
CRL POCUS (mm)	16	12–20	4	100	
CRL vaginal ultrasound (mm)	17.5	14–23	3	100	
CRL difference^d^ (mm)	1	1–2	−6	23	<0.001

^a^CRL = crown-rump length; ^b^POCUS = point-of-care handheld abdominal ultrasound; ^c^CI = confidence interval; ^d^Pairwise comparison of measurement by transvaginal ultrasound versus handheld abdominal POCUS.

In the pilot phase, 13 women were investigated by the senior investigator while training the students, 54 patients were later investigated by the students themselves, and 33 by the senior alone. No patients were falsely identified as having a vital pregnancy (stating FHB + for a pathological pregnancy). Comparing the rate of identifying foetal heart beats in vital pregnancy the senior investigator had an identification rate of 95% (18 out of 19 vital pregnancies) while the students had an identification rate of 77% (37 out of 48 pregnancies), this difference was not statistically significant (*p* = 0.156 Fisher’s exact test).

Interrater correlation was performed where the three examiners independently and blinded determined FHB + versus FHB − on a subset of 19 participants using handheld abdominal POCUS. An excellent Fleiss’ kappa correlation was calculated at 0.93.

## Discussion

### Statement of principal findings

In a prospective setting, we have compared identifying vital intrauterine pregnancy by handheld abdominal POCUS performed by personnel with limited former training (fourth-year medical students) to the gold standard of transvaginal ultrasound by image specialists, for both healthy pregnant women and women with pregnancy complications referred from GPs to a gynaecology outpatient clinic during the first trimester. Handheld abdominal POCUS had no false positives and correctly identified the presence of foetal heart beats with a predictive value of 63% in week 6 and 79% from week 7. CRL measurements were comparable between handheld abdominal POCUS and transvaginal ultrasound with a median difference of 1 mm.

### Strengths and weaknesses of the study

A strength in our study is the blinded setup; using different examiners performing handheld abdominal POCUS and the corresponding transvaginal ultrasound. The difference in the level of experience of the examiners in our study could be considered a strength, as the interrater results were excellent. The lack of experience beforehand for the two students performing the majority of the handheld abdominal POCUS examinations is an indication that limited training is needed to learn how to perform this early pregnancy ultrasound investigation. This leads us to believe that this examination is achievable for most GPs given appropriate training.

The results from this study were meant to be of particular interest to GPs. Handheld abdominal POCUS detecting first trimester vitality is aimed for use in primary health care in symptomatic patients, but our study population, although referred from GPs, was included in specialist care. To test whether this modality (handheld abdominal POCUS) was useful in confirming vitality we needed to examine many presumed normal pregnancies within a limited time-span thus the hospital setting was chosen out of feasibility. The majority of these patients will not be submitted to ultrasound examination by GPs. This may be considered a weakness. However, even in our hospital cohort six (43%) of the 14 patients with suspected pathology (heavy bleeding, abrupt decreasing pregnancy symptoms) were diagnosed with viable pregnancies. This resembles the situation in primary care where nearly 50% of symptomatic patients will be identified with a viable pregnancy [1–3]. This implies that the study results may be considered as valid also in a primary care setting.

Although two of the investigators (fourth-year medical students) had no prior ultrasound experience when the study started, after an introductory phase of 13 examinations, they conducted 54 of the study examinations themselves with a non-inferior detection rate compared to the senior gynaecologist. Although this could imply that the examination could be learned after a limited training period, and as such be suited to learn also for general practitioners, for a GP to achieve a similar experience of >50 examinations of early pregnancies would probably take several years. This must be considered a limitation to our study.

It may also be discussed if the interrater correlation comparisons should have been performed using the whole patient group as originally included, rather than a subset of 19 participants. Performing a repeat but blinded real-time examination by another examiner rather than a video recording might also yield another interrater correlation. In addition to visualization of FHB+, CRL could also have been measured blinded by different examiners.

### Findings in relation to other studies

Two studies (performed in UK and Brazil respectively) have compared the ability to visualize intrauterine pregnancy, foetal heartbeat and CRL using an older model of Vscan (Vscan General Electric Ultrasound^®^) and vaginal ultrasound [11,13]. The Brazilian study investigated 86 women with first trimester bleeding at an emergency gynecology ward where the same experienced specialists performed both abdominal handheld and transvaginal ultrasound [13]. The British study included 101 first trimester pregnant women with suspected complications at an Early Pregnancy and Gynecological Scanning Unit where examiners were blinded regarding results from handheld abdominal POCUS versus the transvaginal ultrasound [11]. Both of these studies found, similar to our study, a good correlation between the two methods, and neither had any false positives regarding visualization of a foetal heartbeat, which had the strongest correlation (Kappa coefficient 0.729 [11] and 0.84 [13].

Neither of these studies specified the ability to visualize FHB + according to gestational length, which was needed within our primary aim to establish the lower limit of gestational length for a reliable vitality confirmation. Separating gestational weeks, we were able to achieve similar good detection rates from week 7 by handheld abdominal POCUS performed by medical students as transvaginal ultrasound performed by image specialists using high-end ultrasound equipment, and a fair detection rate (63% by handheld abdominal POCUS) in week 6.

An increase in BMI had no impact on the ability to visualize FHB + in our study, the same was also noted in the British study [11].

The British study measured a consistent shorter CRL with handheld abdominal POCUS than with transvaginal ultrasound, mean difference of 1.5 mm [11]. This compares well to our result with a median 1 mm shorter CRL measured by handheld abdominal POCUS, which leads to approximately two days difference in estimated gestational length before week 7, and one day after week 7 [5].

A study from the USA compared stationary abdominal ultrasound and vaginal ultrasound in early pregnant women scheduled for medical abortion [15]. CRL measured by abdominal ultrasound was slightly smaller than the transvaginal measurements and led to a median 1.6 days underestimation of gestational length, also supported by studies comparing determination of gestational length with abdominal ultrasound in the emergency unit versus transvaginal ultrasound to be 2–3 days [16,17], this is considered acceptable for clinical use [6]. This indicates that handheld abdominal POCUS has approximately equal visualization ability compared to stationary high-end abdominal ultrasound regarding CRL-measurements.

Although ultrasound is increasingly used in primary health care, not many studies have described the use as a diagnostic tool in early pregnancy. Everett [2] performed a prospective 2-year study from four semirural practices at a health centre in UK. Of 550 confirmed pregnancies, 117 women (21%) experienced bleeding during early pregnancy and 85 of these were investigated by (transabdominal) ultrasound by their GP. For 44 women vital pregnancy was confirmed by the GP ultrasound, while 4 investigations were initially inconclusive and needed a referral for confirmation. This rate of 92% confirmation of vital pregnancies by GPs performing abdominal ultrasound examinations is comparable to our rate of 87% of pregnancies confirmed as vital by handheld POCUS ultrasound examination performed mostly by fourth-year medical students. The study by Everett illustrates that early pregnancy ultrasound may be useful in a GP practise. The volume of possible patients (fertile women) per GP practice will likely be an important factor in considering if early ultrasound examinations should be introduced.

With the aim of developing a basic ultrasound curriculum for primary health care, a Scandinavian Delphi process study set out to identify which ultrasound procedures GPs considered important in their daily work [9]. Of the 30 items that achieved consensus (>67% of participants’ agreement), detecting living intrauterine pregnancy was number tree, first trimester bleeding number 11 and gestational age (CRL measurement) number 14 on their prioritizing list. Thus our study seems timely in addressing that these POCUS examinations may actually be performed using a small hand-held ultrasound device.

### Meanings of the study

This study supports that handheld abdominal POCUS ultrasound may be used as a rule-in examination to confirm a first trimester viable intrauterine pregnancy. If a woman is investigated for suspected pregnancy complications and foetal intrauterine heartbeats are verified, she should be offered reassurance and watchful waiting. The handheld abdominal POCUS is however not sufficient in diagnosing miscarriage or ectopic pregnancy; if a viable intrauterine pregnancy is NOT verified (handheld abdominal POCUS is not conclusive) the woman should be referred to a gynaecological department for further examination.

Further validation studies evaluating actual performance in a primary care setting is recommended. Also determining the necessary amount of training prior to performing clinical examinations is warranted. Based on the findings from this and previous studies, with the aim of increasing skills for future GPs, we have initiated a course at the University of Bergen for last-year medical students teaching handheld POCUS ultrasound in confirming vital intrauterine first trimester pregnancy.
